# Long non-coding RNA *PARTICLE* bridges histone and DNA methylation

**DOI:** 10.1038/s41598-017-01875-1

**Published:** 2017-05-11

**Authors:** Valerie Bríd O’Leary, Sarah Hain, Doris Maugg, Jan Smida, Omid Azimzadeh, Soile Tapio, Saak Victor Ovsepian, Michael John Atkinson

**Affiliations:** 1 Institute of Radiation Biology, Helmholtz Zentrum Munich - German Research Center for Environmental Health, Ingolstaedter Landstrasse 1, 85764 Neuherberg, Germany; 2 Department of Translational Dermatoinfectiology, Westfaelische Wilhelms University Muenster, Faculty of Medicine, Clinical University Muenster, Rontgenstrasse 21, D48149 Muenster, Germany; 3 Department of Pediatrics and Children´s Cancer Research Center, Technical University Munich, Munich, Germany; 4 Institute of Biological and Medical Imaging, Helmholtz Zentrum Munich - German Research Center for Environmental Health, Ingolstaedter Landstrasse 1, 85764 Neuherberg, Germany; 5 Faculty for Electrical Engineering and Information Technology, Technical University Munich, Munich, Germany; 6 Chair of Radiation Biology, Technical University Munich, Munich, Germany

## Abstract

*PARTICLE* (Gene *PARTICL*- ‘*P*romoter of *MAT2A*-*A*ntisense *R*adia*T*ion *I*nduced *C*irculating *L*ncRNA) expression is transiently elevated following low dose irradiation typically encountered in the workplace and from natural sources. This long non-coding RNA recruits epigenetic silencers for *cis*-acting repression of its neighbouring *Methionine adenosyltransferase 2A* gene. It now emerges that *PARTICLE* operates as a trans-acting mediator of DNA and histone lysine methylation. Chromatin immunoprecipitation sequencing (ChIP-seq) and immunological evidence established elevated *PARTICLE* expression linked to increased histone 3 lysine 27 trimethylation. Live-imaging of dbroccoli-*PARTICLE* revealing its dynamic association with DNA methyltransferase 1 was confirmed by flow cytometry, immunoprecipitation and direct competitive binding interaction through electrophoretic mobility shift assay. Acting as a regulatory docking platform, the long non-coding RNA *PARTICLE* serves to interlink epigenetic modification machineries and represents a compelling innovative component necessary for gene silencing on a global scale.

## Introduction

The majority of ribonucleic acids synthesized from the human genome represent long non-coding (lnc) transcripts greater than 200 base pairs. Such lncRNAs have lower expression and greater tissue-specificity compared to messenger RNAs, suggestive of their putative regulatory function^1^. LncRNA plasticity mainly contributes to their capability to interact with diverse biomolecules (DNA, RNA or protein)^2^. This poses a challenge for deciphering the protagonists implicated in lncRNA activities essential for cellular complexity and phenotypic determination.

The lncRNA *PARTICLE* operates an active feedback silencing mechanism upon the putative tumor suppressor *MAT2A* to limit its expression rapidly once *MAT2A* is up-regulated in response to low dose radiation^3^. *PARTICLE* (1432 bp) is transcribed in the antisense direction from within the promoter of *MAT2A*, the product of which encodes the catalytic subunit of methionine adenosyltransferase^4^. *PARTICLE* triplex formation has been demonstrated *in vitro* in the ‘shore’ region of a *MAT2A* promoter CpG island, with evidence found that this lncRNA leads to increased DNA methylation and binds to the Polycomb Repressive Complex 2 (PRC2) subunit Suppressor of Zeste 12 (SUZ12)^3^.

It has emerged that SUZ12 is key for locating the PRC2 catalytic subunit responsible for trimethylation (me3) of histone 3 at lysine 27 (H3K27) during heterochromatin formation^5^. PRC2 also harbors a control module preventing deposition of H3K27me3 on transcriptionally active genes^5^. It has been suggested that focused activity of epigenetic modifiers such as PRC2 and the histone code influence the propensity of an individual gene to become hyper-methylated in malignant tissue, contributing to inactivation of tumor suppressor genes^6, 7^. Histone point mutations can hamper H3K27me3 deposition leading to adverse events such as those implicated during aberrant differentiation of mesenchymal stem cell to skeletal tumorigenesis^8^. Unable to target genomic regions by itself, lncRNAs such as *Xist* (X inactive specific transcript^9^), *Hotair* (HOX transcript antisense RNA^10^), *Meg 3* (Maternally Expressed Gene 3^1^) and *PARTICLE* ^3^ help recruit PRC2 to chromatin.

*PARTICLE* also interacts with G9a (Euchromatic histone-lysine N-methyltransferase 2 (EHMT2)^3^), predicted to maintain a cooperative partnership with DNA methyltransferase 1 (DNMT1) for chromatin binding activity^11^. Recent studies also linking polycomb group repression complexes (including PRC2) to the activity and recruitment of DNA methyltransferase (eg. DNMT1) shed light on possible communication between DNA methylation and histone modifications in the process of gene silencing^12–14^.

In contrast to earlier models that showed *PARTICLEs* repressive ability was, typical of most lncRNAs, restricted to a specific gene at a local (usually in *cis*) level, we now report that *PARTICLE* influences the chromatin methylome via histone modifications and DNMT1 interaction with fundamental implications for epigenetic gene silencing regulation.

## Results

### *PARTICLE* and low dose irradiation act synergistically to enhance the H3K27me3 modification

**Figure 1:**
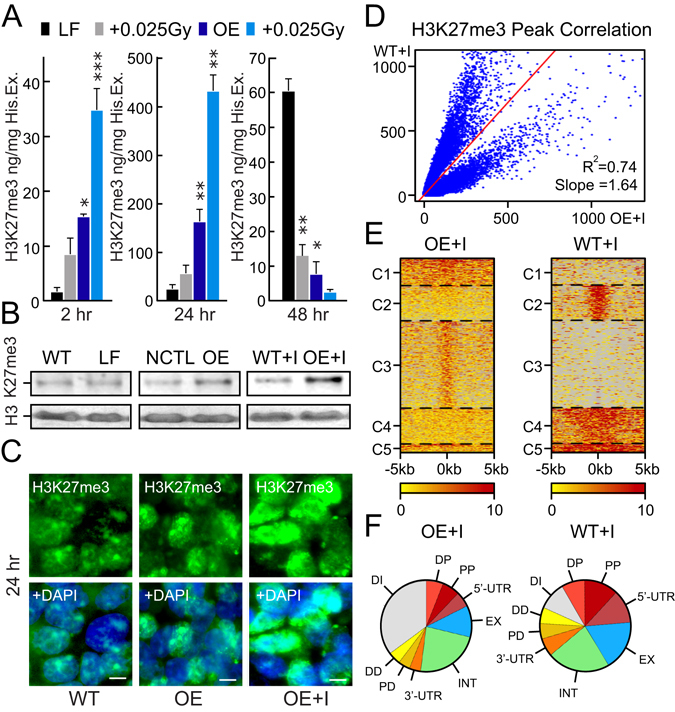
*PARTICLE* and low dose irradiation act synergistically to enhance the H3K27me3 modification. (**A**) Absolute quantification of H3K27me3 per histone extraction concentration (ng/mg His. Ex.) as determined by ELISA 2 hr, 24 hr or 48 hr in MDA-MB-361 transfected with lipofectamine (LF) or *PARTICLE* transcript (over-expression: OE) plus or minus exposure to 0.025 Gy. Data are represented as mean ± SEM and the asterisk(s) represent significant values (p < 0.05). (**B**) Representative Western blots of Histone 3 (H3) and H3K27me3 (upper) in MDA-MB-361 (WT) in LF, NC1 control (NCTL) transfected, OE or OE exposed to 0.025 Gy (OE + I). Cropped images of Western blots shown. (**C**) Epifluorescence micrographs of immunofluoresently detected H3K27me3 (green) in MDA-MB-361 at the 24 hr time point in WT, OE or OE exposed to 0.025 Gy (OE + I). DAPI stained nuclei (blue; merged images below). Scale bar 5 μm. (**D**) Peak correlation scatterplot generated by pairwise comparison of H3K27me3 in irradiated MDA-MB-361 (WT + I) versus irradiated MDA-MB-361 with *PARTICLE* overexpression (OE + I). (**E**) Heatmaps of H3K27me3 distributions across five clustered differentially active regions (C1–C5) in irradiated MDA-MB-361 (WT + I) versus irradiated MDA-MB-361 with *PARTICLE* overexpression (OE + I). (**F**) Pie charts illustrating the genome-wide distribution patterns of H3K27me3 in irradiated MDA-MB-361 (WT + I) and irradiated MDA-MB-361 with *PARTICLE* overexpression (OE + I). DP: Distal Promoter (1–5 Kb); PP: Proximal Promoter (0–1 Kb); 5′UTR: 5′ untranslated region; EX: Exon; INT: Intron; 3′UTR: 3′ untranslated region; PD: Promoter Downstream (0–1 Kb); DD: Distal Downstream Promoter (1–5 Kb); DI: Distal Intergenic Region.

A histone 3 lysine 27 trimethylation (H3K27me3) ELISA revealed augmentation of the H3K27me3 repressive mark within 2 hr post transfection for *PARTICLE* over-expression compared to lipofectamine only controls (LF) (11.9 fold increase; p = 0.0074, Fig. 1A). The H3K27me3 modification level was further substantially augmented in OE versus LF (133.3 fold increase; p = 0.0079) by 24 hr with reduction in this modification at the 48 hr time point following 0.025 Gy irradiation exposure (Fig. 1A). Of note, upregulation of endogenous *PARTICLE* does not occur post 2 hr to 48 hr after such irradiation dosage (manuscript under consideration). This enabled the independent effects of 0.025 Gy irradiation on H3K27me3 to be assessed. Of interest, when *PARTICLE* over-expressing cells were irradiated, H3K27me3 profiles were even more elevated when compared to irradiated LF at 2 hr (23.0 fold increase; p = 0.0003) and 24 hr (239.9 fold increase; p = 0.0024) (Fig. 1A). Western blotting and immunofluorescence analysis revealed a global increase in the H3K27me3 heterochromatin repressive modification in *PARTICLE* over-expressing (OE) cells relative to controls (Fig. 1B,C). A synergistic escalation in this histone modification was apparent in OE 24 hr post very low dose irradiation (0.025 Gy) (Fig. 1B,C). *PARTICLE* has been found to increase expression of EZH2 (Enhancer of Zeste homolog 2), the PRC2 component which catalyzes the addition of methyl groups to histone H3 at lysine 27 (Fig. S4)^15^.

Based on the observation that histone modifications tend to cluster to form domains, a spatial clustering method for the identification of ChIP-enriched regions (SICER)^16^ was utilized to identify signals unlikely to appear by chance. This approach was adopted for the identification of H3K27me3 enriched domains within ChIP-seq datasets. MDA-MB-361 WT (lipofectamine transfected) and *PARTICLE* OE cells 24 hr post exposure to very low irradiation (0.025 Gy) were directly compared. Having passed the quality and purity filter screen (Fig. S1) clear differences emerged. This revealed 24,946 genomic regions with significantly increased H3K27me3 modification in irradiated OE versus WT (Fig. 1D,E). An assessment of average read per million (RPM) signal values was undertaken as quantification of H3K27me3 enriched regions does not typically deliver bell-shaped, symmetrical peaks^17^. The genome-wide distribution patterns of H3K27me3 in irradiated OE versus WT differed considerably with notable increased or decreased presence of this modification in distal intergenic regions or 5′ untranslated regions respectively of the human genome following *PARTICLE* overexpression (Fig. 1F).

### *PARTICLE* alters both the local and global distribution of H3K27me3 in the human genome

**Figure 2:**
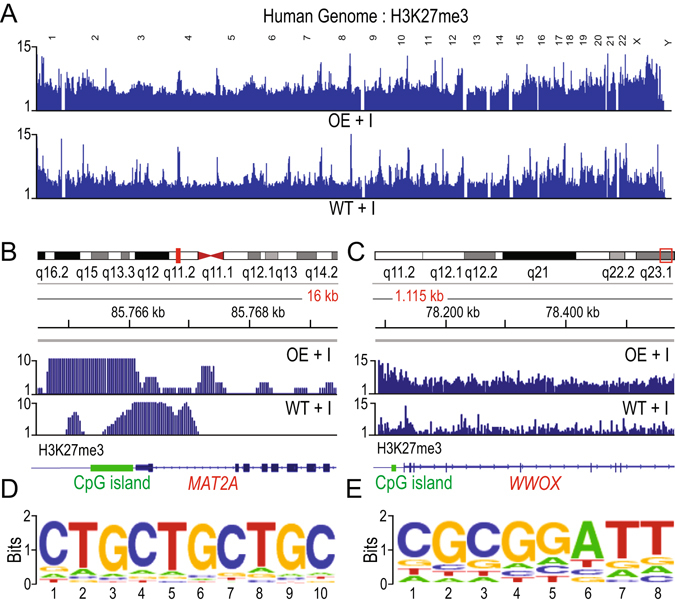
(**A**) Integrative Genomics Viewer screenshot (http://software.broadinstitute.org/software/igv/) of H3K27me3 ChIP-seq track peaks across the human genome (hg 19 assembly) in MDA-MB-361 cells exposed 24 hr previously to 0.025 Gy (WT + I, lower) and over-expressing *PARTICLE* (OE + I, upper). Chromosomal numbers are displayed. (**B,C**) Chromosomal location (red rectangle; top) and integrated genomics viewer screenshots for ChIP-seq data of OE + I (upper) and WT + I (lower). Genomic regions displayed represent *MAT2A* (**B**) and *WWOX* (**C**) loci plus 1.5 kb and 50 kb upstream respectively. Y-axis indicates the read per million (RPM) height of the ChIP-seq readout. CpG island in *MAT2A* (Chr. 2: 85765695–85766983) and *WWOX* (Chr. 16: 78133076–78134066) are depicted as green boxes. (**D,E**) Consensus motif logos within *WWOX* in WT + I (**D**) and OE + I (**E**).

An integrative genomics viewer^18^ enabled virtual H3K27me3 positioning across the human genome to be visualized. ChIP-seq tracking information for irradiated WT and OE revealed an enhancement of this modification in the latter within all autosomal chromosomes. Interestingly, the X-chromosome appeared to have further enrichment of the H3K27me3 modification upon the overexpression of *PARTICLE* (Fig. 2A).

Analysis identified that *MAT2A* and *WWOX* genomic loci (as well as others) were subjected to H3K27me3 repression (Fig. 2B). *PARTICLE* has been reported to increase the methylation of the CpG island 108368 (Chr. 2: 85765695–85766983; NCBI Homo sapien build number 37 version 2) that surrounds the *MAT2A* transcription start site (chromosome 2: 85766100)^3^. ChIP-seq findings revealed a considerable shift in the position and intensity of the H3K27me3 signal upstream of this CpG island (a region of established *PARTICLE* triplex formation^3^) and the *MAT2A* promoter (Fig. 2B). H3K27me3 enrichment along a 1.1 Mb stretch was also found spanning the majority of the *WWOX* locus at two consensus motifs on chromosome 16 in irradiated *PARTICLE* overexpressing (OE) cells versus wild type lipofectamine - only controls (Fig. 2B–E). These results reveal that *PARTICLE* over-expression enhances the histone repressive modification mark across the human genome and specifically within *MAT2A* and *WWOX* tumor suppressor genes.

A predominance of *PARTICLE* triplex binding sites throughout *WWOX* has been identified (manuscript under consideration). Enriched H3K27me3 clustering domains from OE samples were merged with Triplex Domain Finder (TDF) *in silico* data for predicted *PARTICLE* triplex sites within the human genome. This revealed that the *PARTICLE* 627–646 bp domain had significantly higher potential to bind the target H3K27me3 modified domains than randomly chosen similar sized regions from the human genome (806 regions versus 380 regions respectively, p = 0.00001). This would suggest that H3K27me3 modifying enzymes might be guided to specific *PARTICLE* triplex sites to exert their function. INGENUITY integration of ChIP-seq H3K27me3 data with TDF evidence for *PARTICLE* triplex binding sites revealed significant associations with molecular functions eg amino acid metabolism (−log(p-value) = 3.6) and diseases eg cancer (−log (p-value) = 5.2) (Fig. S2).

### *In vivo PARTICLE* interacts with DNA methyltransferase 1 (DNMT1)

**Figure 3:**
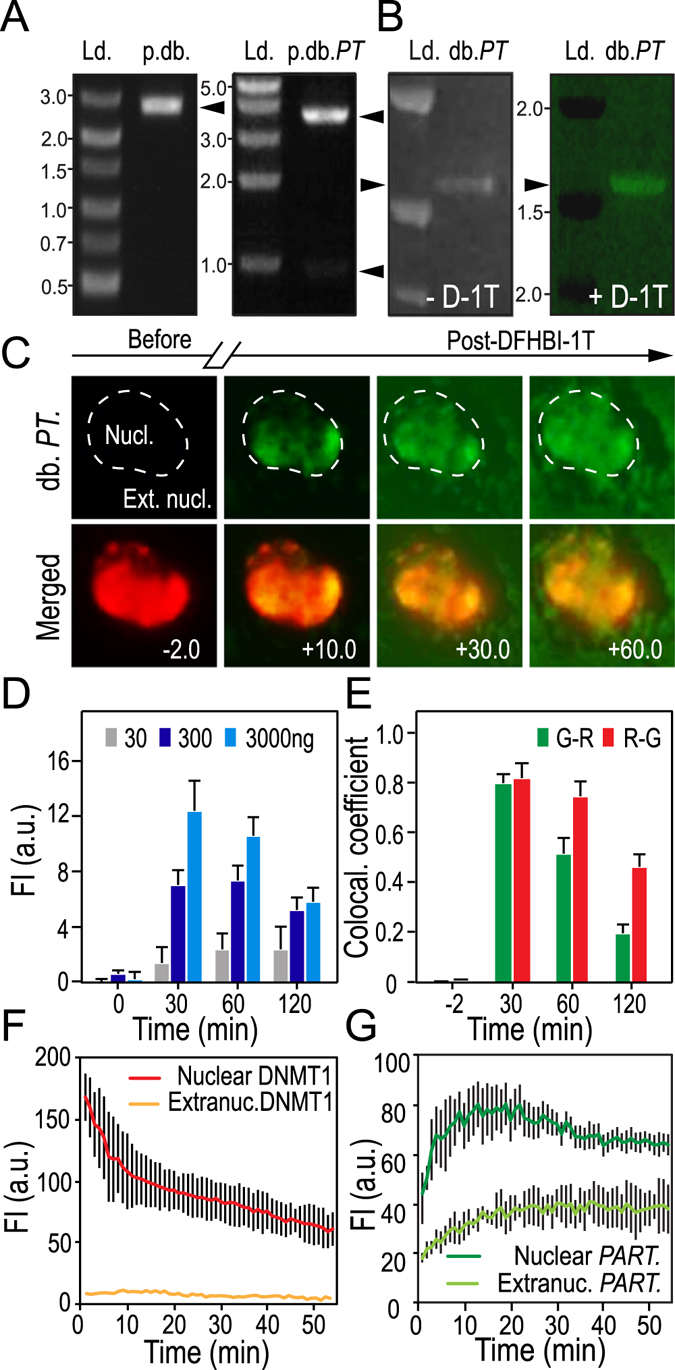
*In vivo PARTICLE* and DNA methyltransferase 1 (DNMT1) interaction. (**A**) Linearized *dbroccoli* in pUC57 (2956 bp; p.db.) after E*coR*I/H*ind*III restriction enzyme digestion resolved on a 1.8% agarose gel (left). Plasmid pGEM-T encoding *dbroccoli*-*PARTICLE* (p.db.*PT*) restriction digested with N*co* I/H*ind*III to produce 3728 bp and 950 bp products (upper and lower arrows). DL3000 (Genscript) and Kb ladder (Ld. left and right gels respectively). (**B**) *dbroccoli*-*PARTICLE* (1678 bp; db.*PT*) *in vitro* transcript resolved through a 12% NuPAGE gel alongside a high range RNA ladder (Ld. Thermo Fisher RiboRuler) before and after staining in DFHBI-1T ((Z)-4-(3,5-difluoro-4-hydroxybenzylidene)-2-methyl-1-(2,2,2-trifluoroethyl)-1*H*-imidazol-5(4*H*)-one; left and right gels respectively). (**A,B**) Cropped images of gels shown. (**C**) Time-lapse fluorescence images of a U2OS cell transfected with db.*PT* and expressing a chromobody to DNMT1-V_H_H fused to TagRFP (DNMT1_RFP) before (minus time point) and after (plus time points) DFHBI-1T (20 μM) addition. Scale bar 2 μm. (**D**) Histograms of arbitrary units (AU) of overall fluorescence intensity with time in U2OS transfected with db.*PT* dose range in the presence of DFHBI-1T (20 μM). Data are represented as mean ± SEM from n = 3 experiments per dose. (**E**) Summary co-localization plots for DNMT_RFP (ChR = red channel) and db.*PT* (ChG = green channel) from ROIs in the nucleus with time. Data are represented as mean ± SEM. (**F,G**) Plots of AU of fluorescence intensity with time from DNMT_RFP (**F**) and in db.*PT* (**G**) in ROIs (n = 10) within nuclear (encircling dashed line) and extranuclear (Extranuc.) cellular compartments.

A dimeric broccoli (dbroccoli) was inserted into a F30 biorthogonal scaffold (246 bp)^19^ and added to the 5′ end of *PARTICLE* RNA (1432 bp). This enabled *in vitro* T7 transcription of dbroccoli-*PARTICLE* (*dbPARTICLE*) chimeric transcripts (1678 bp) that could be visualized following aptamer binding and activation by DFHBI-1T (Fig. 3A,B). Co-transfection of *dbPARTICLE* and a mammalian expression vector encoding chromobody DNMT1-V_H_H fused to TagRFP (DNMT1_RFP) allowed simultaneous live cell imaging of *PARTICLE* and DNMT1 interaction in U2OS (Fig. 3C).

Analysis of the dynamics of DNMT_RFP showed an exponential decline from 160.35 ± 10.47 to 70.87 ± 10.0 arbitrary units (AU) in the nuclear signal (R^2^ = 0.91) over the 2 hr recording period (up to 60 min shown in Fig. 3F). Lower DNMT_RFP signal intensity was evident in the cytosolic/extranuclear compartment, and this also diminished during this time period (10.14 ± 2.0 to 8 ± 0.9 AU, R^2^ = 0.7) (Fig. 3F). In contrast, within 20 min of DFHBI-1T addition, *dbPARTICLE* signal intensity increased in the nucleus from 35.86 ± 9.0 to 80.2 ± 19 AU with a later reduction to 60.15 ± 4.5 AU until 120 min. Under the same conditions, *dbPARTICLE* gradually increased from an intensity level of 19.4 ± 0.5 to 40 ± 11.2 arbitrary units (2 fold increase) in the cytosol within 120 min (up to 60 min shown in Fig. 3G). This finding demonstrates transport of *dbPARTICLE* from the nucleus to the cytosol during this time period. The *dbPARTICLE* was co-localized with DNMT_RFP in the nucleus within 10 min (χ^2^ = 0.87 ± 0.03; Fig. 3C and E). At this time point DNMT_RFP was not solely associated with *dbPARTICLE* (χ^2^ = 0.66 ± 0.02; Fig. 3C and E) as noted by the predominant red signal intensity profiling in the nucleus. By 30 min DNMT_RFP and *dbPARTICLE* showed a strong co-localization in the nucleus (approx. χ^2^ = 0.8; Fig. 3C and E). Independent *dbPARTICLE* in the nucleus became evident after 60 min with diminishing co-localisation signal with time (Fig. 3C and E).

### *PARTICLE* is implicated in global methylome enhancement, *WWOX* CpG island methylation and enzyme activity with DNMT1 interaction

**Figure 4:**
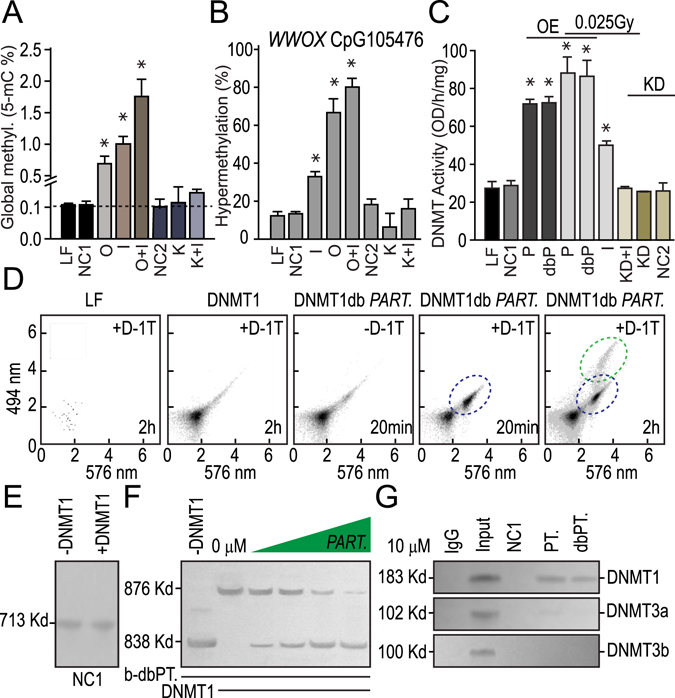
*PARTICLE* is implicated in global methylome enhancement, *WWOX* CpG island methylation and enzyme activity via DNMT1 interaction. (**A**) Histograms of global methylome percentage (5-mC ng/100ng genomic DNA input) from MDA-MD-361 post 24 hr sham irradiation lipofectamine control (LF) with *PARTICLE* over-expression (O) or knockdown (K) and/or 0.025 Gy exposure (I). (**B**) Histograms showing percentage hypermethylation of the CpG island within the *WWOX* promoter in MDA-MB-361 as indicated in (Fig. 2C) above. (**C**) Histograms showing DNMT activity in MDA-MB-361 (nomenclature as indicated in (**A**) above) and with *PARTICLE* (P) and dbroccoli-*PARTICLE* (dbP) overexpression. Data are represented as mean ± SEM (n = 3) and the asterisk(s) represent significant values (p < 0.05). (**D**) Flow cytometry scatter plots of MDA-MB-361 transfected with lipofectamine (LF), DNMT_RFP (DNMT1) and dbroccoli-*PARTICLE* in the absence or presence of DFHBI-1T (20 μM). Time points since DFHBI-1T addition or comparative interval. (**E,F**) Representative nucleotide retardation gels (6%) of electrophoretic mobility shift assay involving binding reactions containing negative *in vitro* transcript control (NC1; E) or biotinylated *dbroccoli*-*PARTICLE* (b-db.*PT*., 10 nM; F), ±human DNMT1 peptide (2.5 μM) and increasing concentrations of unlabeled *PARTICLE*. (**G**) *PARTICLE* (*PT*.) or dbroccoli-*PARTICLE* (*dbPT*.) pulldown in U2OS and immunodetection with anti-DNA methyltransferases. (**E,G**) Cropped images of gels shown.

Global methylome measurements were quantified in MDA-MB-361 over-expressing *PARTICLE* in comparison to lipofectamine only or negative control (NC1) transfected cells (6.1 ± 1 fold increase in the percentage of 5-methylcytosine (% 5-mC), p < 0.05) (Fig. 4A). Exposure of these cells to 0.025 Gy 24 hr previously, demonstrated an even higher degree of global 5-methylcytosine reaching 1 ± 0.02% (p = 0.01) with a synergistic enhancement from irradiation under the same conditions, especially in the presence of *PARTICLE* overexpression (16.29 fold increase compared to LF or negative control, p < 0.05) (Fig. 3A). When compared to controls no significant alteration was found in 5-methylcytosine levels upon *PARTICLE* knockdown or in combination with previous exposure 24 hr earlier to a very low radiation level (Fig. 3A).

*PARTICLE* was found to influence the methylation status of a *WWOX* promoter CpG island (annotated CpG105476) of 990 bp located on chromosome 16: 78133076–78134066 (NCBI *homo sapiens* build number 37/hg19). The transcription initiation site for *WWOX* resides within this region at position chromosome 16: 78133327 orientated in a forward direction (NCBI gene id. 51741). The CpG105476 was hyper-methylated by 13.3 ± 0.9% with the remainder being predominantly unmethylated in sham irradiated lipofectamine transfected MDA-MB-361 cells (LF) (Fig. 3B). Following exposure to a very low irradiation level (0.025 Gy) 24 hr previously, the level of hyper-methylation of CpG105476 increased 1.38 fold to 31.83 ± 3% reflecting methylation events independent of endogenous *PARTICLE* which is not activated after this dosage (Fig. 3B). Nevertheless, in MDA-MB-361 with *PARTICLE* knockdown, the extent of basal CpG10576 hyper-methylation is reduced to 6.25 ± 4% yet increased to 18.19 ± 2% after 0.025 Gy exposure, perhaps reflecting an endogenous influence of this lncRNA on the methylation status of this *WWOX* CpG island. Of interest, over-expression of *PARTICLE* augmented the basal hyper-methylation status of CpG105476 by 4 fold (66.67 ± 7%). Exposure to a very low level of radiation resulted in a further escalation of 5 fold (80.11 ± 3%).

DNMTs transfer methyl groups from s-adenosylmethionine to cytosine to methylate DNA substrate enabling the methylated DNA to be recognized with a 5-methylcytosine antibody. The quantity of methylated DNA which is proportional to enzyme activity was measured thorough ELISA with the activity of the DNMTs proportional to the optical density intensity with time. The significant influence of *PARTICLE* on DNA methyltransferase activity was subsequently revealed providing evidence for the relevance of their interaction. DNMT activity did not differ between controls (p > 0.05) or with *PARTICLE* knockdown (p = 0.19) (Fig. 3C). A significant 1.5 ± 0.3 fold increase in DNMT activity was noted when *PARTICLE* (p = 0.011) or dbroccoli-*PARTICLE* (p = 0.013) were over-expressed in comparison to cells transfected with the negative controls (NC1 or lipofectamine only) (Fig. 3C). Augmentation of DNMT activity was noted in MDA-MB-361 exposed 24 hr previously to a very low irradiation dose (0.8 ± 0.1 fold increase, p = 0.035) (Fig. 3C). Combining *PARTICLE* or dbroccoli-*PARTICLE* overexpression with such irradiation exposure (0.025 Gy) further significantly increased DNMT activity (2 ± 0.4 fold increase, p = 0.022 and p = 0.034 respectively) relative to negative controls (Fig. 3C). Flow cytometric analysis of MDA-MB-361 transfected with *dbPARTICLE* and DNMT_RFP also demonstrated their association within 20 min of DFHBI-1T addition with independent *dbPARTICLE* similarly evident by the 2 hr time point (Fig. 3D). These findings were supported by the association of *dbPARTICLE* with DNMT1 by competitive interaction and gel retardation. An electrophoretic mobility shift of the recombinant human Dnmt1 protein was identified in the presence of biotinylated *dbPARTICLE* plus *PARTICLE* and absent with negative control transcript (Fig. 3E,F). Binding specificity was demonstrated by displacement of the amount of Dnmt1-bound biotinylated *dbPARTICLE* with increasing the concentration of *PARTICLE* (up to 1000 fold molar excess) (Fig. 3F). These data were further supported by immunoprecipitation of DNMT1 with *PARTICLE* in crosslinked cells proving direct interaction of this lncRNA and this DNA methytransferase (Fig. 3G).

Overall, evidence is provided of direct *PARTICLE* DNMT1 interaction that boosts methyltransferase activity levels. Such interaction potentially unveils part of the underlying mechanism by which this lncRNA impacts the global methylome with its notable influence on the CpG island methylation status of the tumor suppressor *WWOX*.

## Discussion

Most recently discovered lncRNAs prevail in the nucleus and tend to be involved in epigenetic regulation^1^. This milieu of lncRNAs either associate with histone or DNA methylation mechanisms^20^. Such double pronged silencing approaches have not been found for a single lncRNA until now. Herein, evidence is provided that *PARTICLE* represents the missing link that unites both histone and DNA methylation. *PARTICLE* is sufficient to affect histone H3K27me3 (via influencing EZH2 expression) throughout the human genome and to enhance this repressive modification mark within the neighbourhood of both the *MAT2A* and *WWOX* tumor suppressor genes. Our findings establish direct interaction between *PARTICLE* and the maintenance DNA methyltransferase DNMT1 coinciding with increased enzyme activity, a global shift in the methylome and an upsurge in *WWOX* CpG island methylation.

The unique ability of lncRNAs to adopt complex secondary and tertiary structures contributes to their greater functional complexity compared to mRNA^21, 22^. While proven for proteins, it is now becoming evident that structure-functional relationships provide important information on lncRNA characteristics for mediating biomolecule interaction in such diverse processes as chromatin organization and transcriptional regulation^22^. *PARTICLE* binds to both the lysine methytransferase G9a and to the SUZ12 component of the polycomb repressive complex 2 (PRC2)^3^. Following low dose irradiation, it could be proposed that *PARTICLE* provides a functional targeting platform enabling specific targeting of otherwise promiscuous repressive modifiers such as PRC2 to chromatin. In keeping with the recognized role of lncRNAs in genomic architectural regulation^23^ and given the interaction between *PARTICLE* and SUZ12^3^, it is tempting to speculate that *PARTICLE* also acts as an epigenetic modifying platform, in this case for PRC2 recruitment to target sites to modulate chromatin structure. Other lncRNAs such as *Xist* and *Firre* (functional, intergenic, repeating RNA element) depend on increased H3K27me3 levels for inactivating and maintaining X chromosome repression^24, 25^. ChIP-seq evidence reported in this study shows H3K27me3 to be generally enhanced throughout the human genome and specifically increased throughout the *WWOX* locus when *PARTICLE* is over-expressed. This data also revealed a shift in the positioning of this modification over the *MAT2A* CpG island, the methylation and expression of which is influenced by *PARTICLE* (Fig. S3)^3^.

Temporary H3K27me3 marks associated with CpG rich genomic regions become replaced by DNA methylation representing a more permanent means of transcriptional repression^26^. Here we show the direct interaction of *PARTICLE* and DNMT1. RNA dependent DNA methylation suggests recognition by DNMT and perhaps recruitment to RNA: DNA: DNA triplexes^27^ such as that formed by *PARTICLE* genome wide (manuscript under consideration). Evidence is presented for enhanced *WWOX* CpG island hyper-methylation with *PARTICLE* over-expression along with synergistic augmentation of this gene repressive modification by very low radiation exposure. Diminished hyper-methylation of this CpG island upon *PARTICLE* knockdown may reflect its inhibitory influence on DNMT1 action with evidence provided here that this lncRNA directly effects the activity of DNA methyltransferases.

Epigenetic mechanisms that incorporate histone modifications, DNA methylation alterations and non-coding RNA expression have been identified as prominent hallmarks for distinguishing physiological from pathological cellular conditions, including tumor suppressor inactivation^28, 29^. Thus, *PARTICLE* may constitute an important bond in the internal crosstalk of the broad language of epigenetics, orchestrating transcriptional silencing of genes including tumor suppressors with wider implications for eliciting carcinogenesis and progression.

*PARTICLE*, a long non-coding RNA, is transcribed in response to irradiation and enables histone modification and DNA methylation to be interwoven. These mechanisms were considered to be relatively independent until now. Operating a double pronged approach enabling methylome repression, *PARTICLE* serves to interlink epigenetic modification mechanisms and represents a compelling innovative component necessary for quelling gene transcription.

## Materials and Methods

### Propagation and maintenance of cell lines

MDA-MB-361 (American Type Culture Collection (ATCC)) was cultivated as previously described^30^. U2OS (ATCC) were grown under similar conditions except Roswell Park Memorial Institute (RPMI) 1640 media (GibcoTM cat # 21875–034) and FBS (10%) were utilized. The identity of all cell lines was verified by microsatellite analysis (Eurofin Medigenomix, Forensik GmbH, Germany). All cultures were routinely checked for mycoplasma contamination using a MycoAlert Mycoplasma detection kit (Lonza, cat. # LT07–218). In general, cells were grown to 80% confluency prior to removal from the dish using trypsin (0.25%)/EDTA (0.02%) and sub-culturing or harvesting.

### Irradiation

All irradiation were performed using a closed HWM-D 2000 Cesium^137^ source (Wälischmiller Engineering GmbH, Markdorf, DE; 10 cm height, 33 cm diameter) at a dose rate of 0.0082Gy/sec. For very low dose irradiation exposure tissue culture dishes were placed into a lead box within the irradiation chamber causing a 10 fold reduction in the dosage rate. Sham irradiation of controls involved only transport to the irradiation facility. Annual calibration was performed by the Helmholtz Zentrum Munich, DE with reference to standards established by the National Physical Laboratory (U.K).

### RNA interference targeting *PARTICLE*

*PARTICLE* knockdown was undertaken with Silencer® Select siRNA interference technology (siRNA id: n307629; Part # 4390771, Thermo Fisher Scientific). MDA-MB-361 cells were grown to 60% confluence and transfected with these siRNAs (10 nM) using lipofectamine as per manufacturer instructions. After 72 hrs, cells were irradiated at 0.025Gy or sham-irradiated (0 Gy). Control conditions included sham irradiation plus transfection with lipofectamine and/or negative siRNA (NC2; cat # AM4615 no.3, Thermo Fisher Scientific). RNA extraction was performed 4 hr and 24 hr post irradiation (or sham irradiation).

### *PARTICLE* overexpression

*PARTICLE* was cloned into the pGEM® - T vector (p.*PART*) (GenScript) and transformed into Top10 bacteria. A colony was grown in ampicillin (100 μg/ml) overnight and plasmid midiprep (Promega) performed. Plasmid concentration and purity was assessed (NanoDrop 1000, Thermo Fisher Scientific) with A260/280 ratio determination with automated sequence validation (GenScript). Plasmid linearization was carried out using 1 μg plasmid DNA and S*ac*I overnight digestion at 37 °C. *PARTICLE* (1432 bp) was *in vitro* transcribed from a pGEM® - T vector (GenScript) using the TranscriptAid T7 High Yield transcription kit (Thermo Scientific, cat # K0441). Transcripts were treated with RNase-free DNase 1 (Thermo Scientific) and purified using an RNeasy mini-elute cleanup kit (Qiagen, cat # 74204) and verified by TBE-agarose (1.8%) electrophoresis. Prior (24 hr) to transfection, MDA-MB-361 or U2OS were seeded (10^5^ cells/35 mm dish) in growth media (described above) in the absence of antibiotic/anti-mycotic to ~70% confluence at the time of transfection. The control template included in the Transcript T7 High Yield Transcription kit (Thermo Fisher Scientific, cat # K0441) as utilised for the production of a 2223 bp ‘run off’ transcript serving as a negative control (NC1) for over-expression studies. Cells were transfected with lipofectamine and *PARTICLE* (4 μg) or negative control (4 μg) as per standard conditions with incubation for 72 hr prior to irradiation exposure.

### Histone 3 lysine 27 trimethylation (me3) immunofluorescence

MDA-MB-361 were cultivated as previously described^3^ on glass coverslips. Having reached ~60% confluence, the media was removed and cells washed two times for 5 min with 1× PBS. Cells were fixed upon exposure to 4% paraformaldehyde for 1 hr and washed for 5 min with 1x PBS. Cells were permeabilized in 1x PBST (1x PBS including 0.5% Triton™ X-100 (Sigma-Aldrich®, cat. # X100–5ML) for 30 min. Following one wash for 5 min in 1x PBS, cells were placed in blocking solution (1x PBS containing 2% goat serum, 5% bovine serum albumin and 0.5% Triton™ X-100) for 1 hr at room temperature. Cells were then exposed to antibody representing rabbit anti-tri-methyl-histone 3 (Lys27) (Thermo Fisher Scientific cat. # PA5-31817, 1: 200 in blocking solution) with o/n incubation at 4 °C. Cells were washed three times for 15 min in 1x PBS and incubated in Alexa fluor® 488 goat anti-rabbit IgG (H + L) (1:500; in blocking solution) for 1 hr at room temperature in the dark. Cells were washed three times for 15 min with 1x PBS and air dried in the dark. To prepare for microscopy, cells on coverslips were mounted in VECTASHIELD™ HardSet™ (Vector; cat. # H1500) containing DAPI, and placed on a glass slide. Results were visualized using an epifluorescence microscope (Zeiss AxioVision).

### Nuclear Isolation

Nuclei were isolated from cell lines (U2OS and MDA-MB-361) using a nuclear extraction kit (Millipore cat # 2900). In brief, cells were grown to 70–90% confluency and removed by trypsinization following standard protocols. Cell pellets (2 × 10^6^ cells) were resuspended in cytoplasmic lysis buffer (500 μl) containing 0.5 mM DTT and protease inhibitor cocktail (1 in 1000 dilution) with incubation on ice for 15 min. Following centrifugation at 250 g for 5 min at 4 °C, the supernatant was discarded and cell pellet resuspended again in cytoplasmic lysis buffer (200 μl). Cell lysis was performed by drawing the cell suspension through a 27-gauge needle. Following centrifugation at 8,000 g for 20 min at 4 °C, the nuclear pellet was resuspended in nuclear extraction buffer (70 μl) containing 0.5 mM DTT and protease inhibitor cocktail (1 in 1000 dilution). Nuclei were disrupted via passage through a 27-gauge needle and incubation for 60 min at 4 °C. Following centrifugation at 16,000 g for 5 min at 4 °C, the supernatant representing the nuclear extract was obtained.

### Histone extraction

This procedure utilized the histone extraction kit (Abcam®, cat. # ab113476). In brief, a pellet was re-suspended in 1x pre-lysis buffer (1 ml), incubated on ice for 10 min and centrifuged for 1 min at 7800 × g at 4 °C. The cell pellet was re-suspended in lysis buffer (200 μl), incubated on ice for 30 min and centrifuged for 5 min at 11300 × g at 4 °C. The supernatant was transferred into a fresh tube and mixed with balance - DTT buffer (70 μl). Total histone protein concentration was measured using a bicinchoninic acid assay (Thermo Fisher Scientific™ Pierce; cat. # 23225).

### Detection of Histone 3 and lysine 27 trimethylation (H3K27me3) and EZH2 by Western blotting

Nuclear extracts or cell lysates (25 μg/lane) were loaded in 1x NuPage loading buffer (Thermo Fisher Scientific, cat # NP0007) onto 12% NuPage® Bis-Tris gels (Thermo Fisher Scientific, cat # NP0336BOX) alongside a Precision Plus Protein dual colour standard marker (Bio-Rad, cat # 1610374). Following standard electrophoresis using 1x MOPS running buffer and transfer, nytran membranes were incubated for 1 hr in blocking solution (TBST, 5% BSA) and probed overnight at 4 °C with mouse monoclonal anti-Histone H3 (abcam, cat # ab10799, 1 in 1000 dilution) or rabbit polyclonal anti-H3K27me3 (Thermo Fisher Scientific, cat # PA5-31817, 1 in 1000 dilution). Detection of EZH2 was performed using rabbit monoclonal anti-EZH2 (Cell Signaling Technology, cat # 5246, 1 in 500 dilution) with normalization against the endogenous control GAPDH (Santa Cruz, cat # sc-47724, 1 in 1000 dilution). Following extensive washes in 1x TBST, membranes were incubated in goat anti-mouse or rabbit IgG –alkaline phosphatase (Sigma Aldrich, 1 in 10000 in blocking solution). Membranes were washed 3 times for 15 min in TBST, followed by signal development in BCIP/NBT liquid substrate (Sigma Aldrich, cat # B1911) with image recording using an Alpha Innotech gel imager (Fluor Chem ® HD2, BioZym), n = 3.

### ELISA H3K27me3 quantification

This procedure utilized the histone 3 (tri-methyl K27) quantification fluorometric kit (abcam®, cat. # ab115073). In brief, a standard curve was established using a kit included standard control (H3K27me3, 100 μg/mL) diluted with antibody buffer to provide a concentration range from 1.5–100 ng/μl. Histone extract (100 ng) was deemed optimal for determining modification levels within the standard curve range. Antibody buffer (50 μl) was added to standards and samples in a 96 well transparent dish (Thermo Fisher Scientific™, cat. # 168136). The plate was covered with parafilm, mixed very gently and incubated for 2 hr at room temperature. Wells were aspirated and washed three times with 1x wash Buffer (150 μl). Detection antibody (1:1000, 50 μl) was added to each well and incubated for 60 min at room temperature on an orbital shaker at 0.7 × g. Wells were aspirated and washed with 1x wash buffer (150 μl, X 6). Away from light, pre-mixed fluoro-development solution (50 μl) was added into each well and incubated for 5 min at room temperature. Fluorescence signal was measured at an excitation wavelength of 530 nm and an emission wavelength of 590 nm using a Tecan Infinite® M200.

### Chromatin immunoprecipitation

MDA-MB-361 cells treated with lipofectamine (WT) and/or *PARTICLE* over-expression were exposed to 0.025 Gy (as described above). After 24 hr 1 × 10^8^ cells were formaldehyde cross linked according to the published protocol^31^. Cells were sonicated using the following conditions: sonication level = 3, duration time = 5 min with 30 seconds on/off mode, without probe contact with the 5 ml eppendorf tube (Sonfier B-12; Branson sonic power company). Genomic DNA fractionation was assessed by 1.5% TBE/agarose gel electrophoresis. Dynabeads sheep anti-rabbit IgG (Life Technologies, cat # 11203D) were placed in blocking solution (1x PBS including bovine serum albumin (0.5%)) at 4 °C and collected on a magnetic rack. Following washes (X 20) in blocking solution, beads were re-suspended in blocking solution (250 μl) containing rabbit anti-tri-methyl-histone 3 (Lys27) (anti-H3K27me3 (10 μg), Thermo Fisher Scientific cat. # PA5-31817) with o/n incubation on a rotator at 4 °C. Anti-H3K27me3/dynabead mix was added to the sonicated cell lysate with o/n incubation at 4 °C. Further washing, elution and crosslinking reversal were performed as previously described^31^. DNA purification was carried out using a Maxwell® 16 LEV Blood DNA kit (Promega cat # AS1290) and Maxwell® 16 machine. DNA quantification was determined using a Qubit® dsDNA high sensitivity assay kit (Invitrogen) with integrity (quality and fragment distribution) tested on a 2100 Bioanalyzer (Agilent Technologies). Illumina TruSeq ChIP library preparation and data analysis was outsourced to IMGM laboratories (Munich, Germany) and Active Motif (CA, USA) respectively. ChIP-seq track comparison was performed using the Integrative Genomics Viewer online software from the Broad Institute^18^.

### Tagging dbroccoli aptamer to *PARTICLE*

RNA aptamers resembling the fluorophore in GFP have been designed^32^ and optimized^33^. Broccoli is an enhanced tag for imaging RNA in mammalian cells that exhibits green fluorescence upon binding DFHBI-1T ((Z)-4-(3,5-difluoro-4-hydroxybenzylidene)-2-methyl-1-(2,2,2-trifluoroethyl)-1*H*-imidazol-5 (4*H*)-one). Dimeric Broccoli (dBroccoli) contains two broccoli aptamers within one long stem-loop. A biorthogonal scaffold (F30) which reportedly enhances broccoli fluorescence *in vivo* and contains two entry points for dBroccoli insertion has been used for the creation of the F30-2xdBroccoli tag^19^. Using the online sequence information (http://www.jaffreylab.org) the F30-2xdBroccoli tag was synthesized and cloned into pUC57 (p.db.) and 5′ subcloned into p.*PART* (p.db.*PT*) (GenScript, clones available upon request). Plasmid preparation, automated sequencing (Eurofins) followed by restriction digestion (as indicated in the figure legend) and electrophoresis confirmed the correct identity and orientation of the clones. *In vitro* dbroccoli-*PARTICLE* (db.*PT*, 1678 bp) synthesized as described above were specifically visualized by 12% NuPAGE and post staining in DFHBI-1T (10 μM; in HEPES pH 7.4 (40 mM), KCl (100 mM), MgCl_2_ (1 mM)) with gel image visualization using 488 nm excitation. Following extensive washes with water (5 × 5 min) the gel was post stained with ethidium bromide (5 μl of 10 mg/ml stock in 100 ml dH_2_0) for visualization of RNA transcript and ladder (Thermo Fisher Scientific, RiboRuler High Range cat # SM1823). U2OS were transfected using lipofectamine (Invitrogen) as per manufacterer’s instructions with a dose range of db.PT (30 ng–3000 ng). Dose response of *dbPARTICLE* determined cellular sensitivity to 3 μg dbPARTICLE/10^5^ cells while 300 ng dbPT/10^5^ cells proved optimal for image acquisition (Fig. 3D). In the presence of DFHBI-1T (10 μM) live image acquisition was undertaken for 2 hr in an environmental chamber (5% CO_2_, 10% O_2_) using an inverted Axiovert 200 (Zeiss) fluorescence microscope with apotome slide module activation.

### Dnmt1 chromobody®-TagRFP plasmid and dbroccoli-*PARTICLE* co-transfection and live imaging

The Dnmt1 chromobody**®-**TagRFP plasmid (pDC-TagRFP, Chromotek) is a mammalian expression vector encoding the epigenetic DNA (cytosine-5)-methytransferase 1 marker Dnmt1-VHH fused to the red fluorescent protein TagRFP (from Evrogen). The vector backbone contains immediate early promoter of cytomegalovirus (CMV) for protein expression, SV40 T-antigen, CoIE1 origin of replication for propagation in E.*coli* and f1 origin for single-stranded DNA production. SV40 polyadenylation (SV40 poly A) direct proper processing in the 3′- end of the reporter mRNA. SV40 early promoter (Psv40) provides neomycin resistance gene (Neo) expression to select stably transfected eukaryotic cells using G418. The bacterial promoter (P) provides kanamycin resistance gene expression (Kan’) in E.*coli*. U20S were seeded 24 hr prior to transfection (2 × 10^5^/35 mm dish) in antibiotic/antimycotic free growth media (for conditions see above). Cells were co-transfected using lipofectamine (Invitrogen) as per manufacturer’s instructions with pDC-TagRFP (3 μg) and db.*PT* (300 ng). After 24 hr, in the presence or absence of DFHBI-1T (10 μM) live cell imaging was undertaken for 2 hr at 2 min intervals in an environmental chamber (5% CO_2_, 10% O_2_). Signal bleaching restricted recording to 2 hr rather than overnight. Images were acquired using a GFP and TexasRed filter wheel on an inverted Axiovert 200 (Zeiss) fluorescence microscope with apotome slide module activation. Colocalisation and relative intensity analysis were performed with FIGI software (NIH).

### Flow cytometry

MDA-MB-361 (10^3^ cells) were resuspended in 1 X PBS (100 μl) in a polypropolyene tube followed by flow cytometry in an BD Accuri™ C6 Plus platform (BD Biosciences). FITC (494 nm) and PE (576 nm) excitation filter wheels were selected for cellular scatter plot visualisation without gating.

### Electrophoretic mobility gel shift assay (EMSA)

Binding reactions (20 μl) were set up that included biotin end-labelled (Thermo Fisher Scientific, cat # 20160) db.*PT* transcripts or negative transcript (NC1, see above) (10 nM), ± DNMT1 peptide (2 μg, Abcam, cat # ab152344), ± unlabelled *PARTICLE* as competitor (1–10 μM) in REMSA binding buffer (1X, HEPES (10 mM) pH 7.3, MgCl_2_ (1 mM), DTT (1 mM)), glycerol (50%) plus KCl (6.25 mM) and incubated at RT for 30 min. Loading buffer (5 μl, 6X, 15% Ficoll 400, 0.25% bromophenol blue, 0.25% xylene cyanol, 1X TBE) was added to each sample before loading onto a pre-cast nucleotide retardation gel (6%, Life Technologies, cat # EC63652BOX). The gel was pre-electrophoresed in TBE (0.5X) running buffer for 30 min prior to sample loading. Electrophoresis was carried out at 4 °C at 100 V followed by sample transfer to nytran membrane and UV cross-linking (120 mJ/cm^2^; 254 nm, 60 seconds). Biotinylated *PARTICLE* was detected by exposure to streptavidin-HRP and chemiluminescence detection (Thermo Scientific, cat # 89880).

### Crosslinking and RNA pull-down

*PARTICLE*, dbroccoli *PARTICLE* and negative control (NC1*)* were *in vitro* transcribed and biotin end-labelled (Thermo Fisher Scientific, cat # 20160) with T4 RNA ligase. Transcripts were treated with RNase-free DNase 1 (Thermo Fisher Scientific) and purified using an RNeasy mini-elute cleanup kit (Qiagen, cat # 74204). Nuclear proteins were extracted (Abcam, cat # ab113474) from U2OS. Nuclear lysate (1 mg) were incubated with purified biotinylated transcripts (3 μg) for 1 hr at 25 °C and UV crosslinked for 2 min at 0.125 joules; IgG control antigen or complexes were isolated with streptavidin agarose beads (200 μl, Thermo Fisher Scientific, cat # 20353). Following extensive washes (1x PBS, 4 times), biotinylated transcripts with bound complexes were eluted by incubation in SDS-PAGE sample buffer (70 °C) followed by centrifugation at 14,000 g for 5 min. Input and supernatant samples were loaded onto 12% Bis Tris NuPage gels, transferred to nytran membranes and probed with anti-DNMT1 (Chromotek, cat # 2E8-125), anti-DNMT3a (Abcam, cat # 2850) or anti-DNMT3b (Abcam, cat # 13604) and anti-rabbit or anti-mouse AP secondary antibodies. Detection was performed as described above.

### Analysis of DNA methylation status of the CpG island in the *WWOX* promoter

Genomic DNA was isolated from MDA-MB-361 (conditions: LF, OE, NC1, KD, NC2) 24 hr post irradiation (0.025 Gy) or sham-irradiation. Genomic DNA was digested using methylation-dependent and sensitive restriction enzymes using an EpiTect Methyl II DNA restriction kit (Qiagen, cat. # 335452) as per manufacturers’ instructions and assessed by SYBR green real-time PCR detection using the EpiTect Methyl II PCR primer assay for *WWOX* (CpG island 105476): (Qiagen, cat. # EPHS105476-1A). To accurately measure the relative percentage of unmethylated and methylated DNA within the CpG island region an online analysis tool (http://www.sabiosciences.com/dna_methylation_data_analysis.php) was utilized.

### Global methylome determination

Genomic DNA (100 ng) was isolated from MDA-MB-361 (conditions: LF, OE, NC1, KD, NC2) 24 hr post irradiation (0.025 Gy) or sham-irradiation using a Maxwell® 16 LEV Blood DNA kit (Promega cat # AS1290) and Maxwell® 16 machine. The global DNA methylation status was determined using a colorimetric MethylFlashTM methylated DNA quantification kit (Epigentek, cat # P-1034) as per manufacturer’s instructions.

### DNMT activity determination

Quantification of DNA methyltransferase activity was performed and analysed according to manufacturer’s instructions using a colorimetric kit (Abcam, cat # ab113467) and nuclear extracts isolated (described above) from MDA-MB-361 (conditions: LF, *PARTICLE* OE, dbroccoli-*PARTICLE* OE, NC1, KD, NC2).

### Statistical Analysis

Values in the text are expressed as the mean ± S.E.M., and n refers to the number of independent biological relicated data, n = 3. Groups were tested using the Student’s t-test with p values < 0.05 taken to indicate statistical significance. Co-localization testing was assessed using FIGI software (NIH).

### Meta-analysis

INGENUITY pathway analysis (IPA) (http://www.ingenuity.com) was utilised to analyze H3K27me3 ChIP-seq data related to *PARTICLE* over-expression (Fig. S1). IPA enabled the visualisation of changed molecular functions and disease risk with associated significance expressed as −log of the calculated p-value (p < 0.05 equivalent to −log = 1.3).

## Electronic supplementary material

Supplementary Information
