# Supplementary material for: Long non-coding RNA PARTICLE bridges histone and DNA methylation
Source: Sci Rep. 2017 May 11;7:1790. doi: 10.1038/s41598-017-01875-1 (PMC5431818; doi:10.1038/s41598-017-01875-1)
Supplement: Supplementary file 1 — Supplementary Information [file 41598_2017_1875_MOESM1_ESM.doc]

**Supplemental Information**

**Long non-coding RNA *PARTICLE* bridges histone and DNA methylation.**

**Valerie Bríd O'Leary, Sarah Hain, Doris Maugg, Jan Smida, Omid Azimzadeh, Soile Tapio, Saak V. Ovsepian, Michael John Atkinson.**


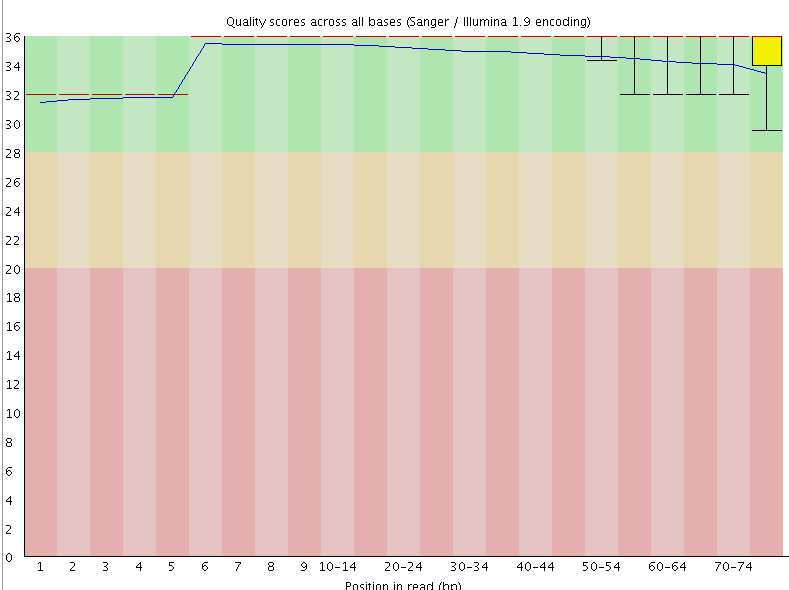

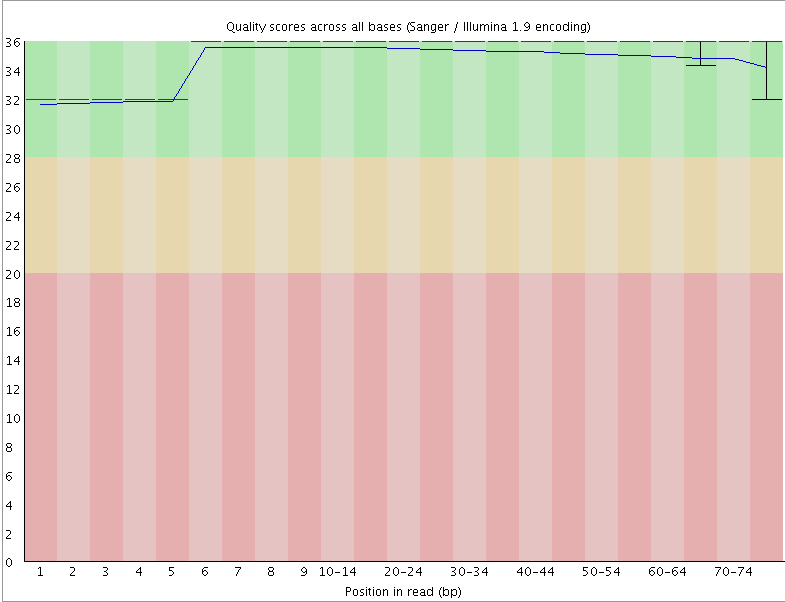


***PARTICLE* - OE + 0.025 Gy**

***PARTICLE - WT* + 0.025 Gy**

**Quality scores across all bases**

**(Sanger / Illumina v 1.9)**

**Quality scores across all bases**

**(Sanger / Illumina v 1.9)**

**36**

**34**

**32**

**30**

**28**

**26**

**24**

**22**

**20**

**18**

**16**

**14**

**12**

**10**

**8**

**6**

**4**

**2**

**0**

**36**

**34**

**32**

**30**

**28**

**26**

**24**

**22**

**20**

**18**

**16**

**14**

**12**

**10**

**8**

**6**

**4**

**2**

**0**

**1 2 3 4 5 6 7 8 9 10-14 20-24 30-34 40-44 50-54 60-64 70-74**

**1 2 3 4 5 6 7 8 9 10-14 20-24 30-34 40-44 50-54 60-64 70-74**

**position in read (base pair)**

**position in read (base pair)**

**Fig. S1 is relevant to Fig. 1 and 2.** ChIP-seq analysis of histone 3 lysine 27 trimethylation (H3K27me3) in irradiated MDA-MB-361 in the presence/absence of *PARTICLE* over-expression. Assessment of ChIP-seq sample quality, represented here as the sequence quality score versus base pair position using fastqc. This is regarded as a critical initial step in ChIP-seq data analysis and a requirement for appropriate interpretations of subsequent downstream analyses ^1^. These representative images indicate good quality data, given the low error rate and variability of the reads.

**Fig. S2 related to Fig. 1**. Ingenuity software (Qiagen) analyis integrating *PARTICLE* triplex binding sites and H3K27me3 Chip-seq data. (A - B) Diseases (A) and molecular functions (B) significantly associated with *PARTICLE* triplex binding sites plus enriched H3K27me3 regions in the human genome. Histogram height reflects the associated -log of the calculated p-value, whereby a significant p value of p < 0.05 is equivalent to -log = 1.3.


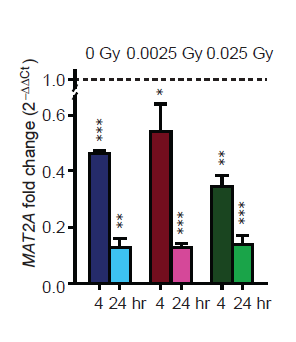


**Fig. S3 related to Fig.2.** Histograms of *MAT2A* expression 4 or 24hr following 0, 0.0025 or 0.025 Gy irradiation in MDA-MB-361 (over-expressing *PARTICLE* following *in vi*tro transfection). Values were normalized with the TATA-binding protein (TBP) encoding endogenous gene with relative expression comparison to sham-irradiated (0 Gy) equivalent cells (dashed lines). Data are represented as mean ± SEM with significance represented by asterisks (p < 0.05) where appropriate.


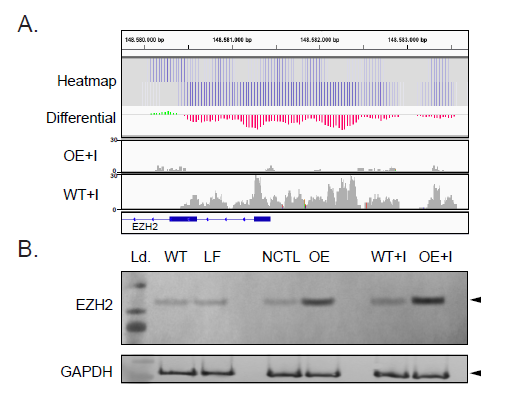


**Fig. S4 related to Fig.2.** (A) Heatmap of H3K27me3 tract intensity (upper: MDA-MB-361 24 hr post very low dose irradiation exposure (WT + I; bottom) ± *PARTICLE* overexpression (OE + I; top); Differential data represents the subtraction of H3K27me3 tract data of I minus OE + I; Integrative Genomics Viewer screenshot of H3K27me3 ChIP-seq track peaks across chromosome 7: 148,580,000 – 148,583,750 encoding the 5’ upstream region of Enhanzer of Zeste Homolog 2 (EZH2). (B). Representative Western blots of EZH2 and GAPDH in MDA-MB-361 (WT) cell lysates in LF, NC1 control (NCTL) transfected, WT or OE exposed to 0.025 Gy (WT + I or OE + I, respectively). Gels are cropped images. Precision plus protein standard (Bio-Rad, cat. # 161 – 0374; Ld.) loaded on the left side of both representative gels. Arrow indicate the position of bands of interest.

**References**

1 Maze, *I. et a*l. Analytical tools and current challenges in the modern era of neuroepigenomics*. Nat Neuros*c**i** 17, 1476-1490, doi:10.1038/nn.3816 (2014).
